# Age and kidney function are the primary correlates of fasting plasma total homocysteine levels in non-diabetic and diabetic adults. Results from the 1999–2002 National Health and Nutrition Examination Survey

**DOI:** 10.1186/1743-7075-2-13

**Published:** 2005-05-26

**Authors:** Glen E Duncan, Sierra M Li, Xiao-Hua Zhou

**Affiliations:** 1 Department of Epidemiology, Interdisciplinary Graduate Program in Nutritional Sciences, University of Washington, Seattle, WA 98195, USA; 2 Department of Biostatistics, University of Washington, Seattle, WA 98195, USA; 3 HSR&D Center of Excellence, VA Puget Sound Health Care System, Seattle, WA 98101, USA

## Abstract

**Background:**

Plasma total homocysteine (tHcy) is commonly elevated in persons with diabetes. This may be due to effects of insulin and/or glucose and/or metabolic control on the metabolism or plasma levels of tHcy. This study examined the effects of fasting plasma glucose status on fasting tHcy levels among adults without diabetes, and diabetes *per se *among adults with a self-report history of diabetes.

**Methods:**

Analysis of data on adults (≥ 20y) who had fasted at least 8 hours, from the National Health and Nutrition Examination Survey (1999–2000 and 2001–2002). Subjects with no self-report history of diabetes were grouped according to fasting plasma glucose status as normal (< 100 mg/dL = NFG, *n *= 2,244), impaired (≥ 100 < 126 mg/dL = IFG, *n *= 1,108), or a provisional diagnosis of diabetes (≥ 126 mg/dL = DFG, *n *= 133). Subjects with a self-report history of diabetes (*n *= 275) were examined separately.

**Results:**

Fasting tHcy was higher (*Ps *< 0.01) among non-diabetic subjects with DFG and IFG, compared to NFG (median [95% confidence interval] = 8.6 [8.0–9.2], 8.3 [8.1–8.5], and 7.4 [7.3–7.5] μmol/L, respectively). Diabetic subjects had levels similar to non-diabetic subjects with DFG and IFG (8.3 [7.9–8.6] μmol/L). Age and estimated creatinine clearance were strong correlates of fasting tHcy among non-diabetic subjects (*r *= 0.38 to 0.44 and *r *= -0.35 to -0.46, respectively) and diabetic subjects (*r *= 0.41 and *r *= -0.46, respectively) (*Ps *< 0.001), while fasting glucose and glycohemoglobin (HbA_1c_) were weaker (but still significant) correlates of tHcy in non-diabetic and diabetic subjects. Fasting glucose status was not a significant independent predictor of fasting tHcy levels in non-diabetic subjects, and HbA_1c _was not a significant independent predictor of tHcy in diabetic subjects (*Ps *> 0.05).

**Conclusion:**

Fasting tHcy levels are elevated among non-diabetic adults with elevated fasting glucose levels, compared to persons with normal fasting glucose levels, and among diabetic adults. However, elevations in fasting tHcy appear to be mediated primarily by age and kidney function, and not by measures of glucose metabolism.

## Background

Clinical studies have established that individuals with diabetes have a two- to six-fold increased risk for various manifestations of cardiovascular disease (CVD), compared to age matched nondiabetic subjects [1-3]. Although individuals with diabetes have a higher prevalence of traditional CVD risk factors (e.g., hypertension and dyslipidemia) compared to nondiabetics, these risk factors do not fully account for the excess mortality associated with diabetes [4]. Even before the clinical diagnosis of type 2 diabetes, a significantly elevated risk of CVD was found in a large cohort of women in the U.S. who subsequently developed diabetes compared to women who remained nondiabetic [5]. Thus, having diabetes is an established independent risk factor for the development of CVD.

In an early meta-analysis assessing the relationship between plasma total homocysteine (tHcy) and CVD [6], it was reported that elevated tHcy was strongly and independently related to several manifestations of CVD in the general population. Subsequent meta-analyses have confirmed the significant relationship between tHcy and CVD risk in healthy populations [7,8], although the strength of association in these studies was weaker than reported previously. These meta-analyses were based on studies in healthy populations, thus, the relationship between tHcy and CVD risk in subjects with a disease highly related to CVD risk itself, such as diabetes, is largely unknown. In a large cohort of 50–75y men from the Netherlands [9], the risk for any CVD was increased with a parallel elevation in tHcy across subjects stratified by glucose tolerance – from normal glucose tolerance to impaired glucose tolerance to type 2 diabetes – even after adjustment for traditional CVD risk factors and serum creatinine. In a prospective analysis of this cohort, elevated tHcy was related to 5-year mortality [10] and coronary events [11] in subjects with type 2 diabetes, independent of other CVD risk factors. In these studies, elevated tHcy was a stronger risk factor for CVD in diabetic than in nondiabetic individuals.

It is unknown why tHcy is elevated in persons with diabetes. Elevated tHcy was more common in patients with complications of type 2 diabetes, compared to patients with uncomplicated disease and controls [12]. Increased tHcy was also reported in patients with poorly controlled type 2 diabetes, compared to well controlled patients and matched controls in a clinic based study in Poland [13]. Results of a recent prospective study in an older cohort of Italian adults [14] demonstrated that tHcy was decreased in patients with type 2 diabetes who had a modest improvement in metabolic control (assessed by glycohemoglobin, HbA_1c_), whereas tHcy increased in subjects who had an increase in HbA_1c _and was unchanged in patients who had no change in HbA_1c_. Poor metabolic control was also associated with elevated tHcy levels in patients with type 1 diabetes [15], consistent with studies in which lower tHcy levels were reported for well controlled, insulin-treated type 1 diabetics than in controls [16]. Together, these studies suggest that tHcy levels in persons with diabetes may be at least partially related to metabolic control. Similarly, *in-vitro *evidence demonstrates that important enzymes of intracellular homocysteine metabolism were directly regulated by insulin and glucose concentrations [17]. This study tested the hypothesis that fasting plasma glucose status independently predicts fasting tHcy in a large, representative sample of non-diabetic U.S. adults. This study also examined correlates of fasting tHcy among a large sample of U.S. adults with a self-report history of diabetes.

## Methods

This report is based on data from the combined 1999–2000 and 2001–2002 National Health and Nutrition Examination Survey (NHANES). The study design is a stratified, multistage probability sample of the civilian non-institutionalized U.S. population. Approximately 9,965 persons aged 2 months to 85 years were studied in NHANES 1999–2000, and 11,039 in NHANES 2001–2002. A sub sample of over 3,000 individuals from each survey was invited to attend a morning examination after having fasted overnight. The data collection procedure involved an initial home interview component comprised of a screener, sample person, and family interview questionnaire. All interviewed persons were then invited to complete the health examination component of the survey in a mobile examination center, which included a series of health questionnaires, a physical examination, and a laboratory component. Fully informed consent was obtained from all participants as approved by the National Center for Health Statistic's Institutional Review Board.

Details of the NHANES protocol are available elsewhere. Briefly, height was measured in an upright position with a stadiometer, and weight at a standing position on a self-zeroing scale. Blood pressure measurements were performed by trained technicians using a standardized protocol. Three and sometimes four measurements were made on all subjects with a mercury sphygmomanometer, and the first and fifth Korotkoff sounds were recorded to represent the systolic and diastolic pressures. We used the average of three recorded measurements in all data analyses. Blood analytes were stored under appropriate frozen conditions until they were shipped to a central laboratory for analysis. Plasma tHcy was measured using a flourescence polarization immunoassay (Abbott Diagnostics). Plasma glucose was measured using an enzymatic technique employing the hexokinase/glucose-6-phospate dehyrogenase reaction. Glycohemoglobin was measured in whole blood using a fully automated glycohemoglobin analyzer (Primus Instruments) and boromate affinity high-performance liquid chromatography. Total cholesterol was measured in serum or plasma using an enzymatic technique. Serum levels of folate and vitamin B_12 _were measured using a radioassay kit (Bio-Rad Laboratories), and serum creatinine was measured using a photometric method employing the Jaffe reaction. Creatinine clearance was estimated (**C_Cr_**) using the Cockcroft-Gault formula [18].

Data from each survey were linked using the unique survey participant identifier (SEQN). The primary analyses consisted of 3,485 adults (20–85y) with a fasting (at least 8 hours) plasma glucose value recorded and who responded "No" to a lead-in question on diabetes history (see below). Subjects with a fasting plasma glucose < 100 mg/dL were categorized as having normal fasting glucose (NFG), those with a value ≥ 100 < 126 mg/dL as having impaired fasting glucose (IFG), and those with a level ≥ 126 mg/dL as having a provisional diagnosis of diabetes (DFG) [19]. The second set of analyses consisted of 275 adults (20–85y) based on a personal interview on diabetes, including use of medications and symptoms associated with diabetes. All subjects were asked a lead-in question pertaining to history of diabetes ("Have you ever been told by a doctor or health professional that you have diabetes or sugar diabetes?"). Subjects with a missing response, those who refused to respond, and those who responded "No", "Don't know", or "Borderline" were excluded from these analyses. Pregnant females were also excluded from both sets of analyses.

### Statistical Analysis

Data were analyzed using SAS (Version 9.1) survey procedures. Both analyses used the four-year fasting weights (WTSFA4YR) to estimate means and 95% confidence intervals, and the masked variance units (pseudo-primary sampling units [SDMVPSU] and pseudo-stratum [SDMVSTRA]) to estimate standard errors of those means. Continuous variables were analyzed using PROC SURVEYMEANS and are presented as a mean (± standard error). Categorical variables were analyzed using PROC SURVEYFREQ and are presented as a frequency (*n*), weighted frequency (based on sampling weights), and proportion [with 95% confidence interval]. Proportions are based on a percentage of the weighted frequencies. Differences in continuous variables were tested univariately using the *t*-test for independent samples (using PROC SURVEYREG), and prevalence values for categorical variables were compared using the χ^2 ^test for proportions (in PROC SURVEYFREQ). Statistical significance was established at α = 0.05 *a priori*, and multiple comparisons were adjusted using the Bonferroni method.

## Results

The primary analyses included a sample of 3,485 adults. By definition (see *Methods *section), none of these subjects had a self-report history of diabetes, and none reported taking insulin or diabetic pills to lower blood sugar. The sex distribution was roughly 50% male and female, and race distribution 24% Mexican-American, 5% other Hispanics, 51% non-Hispanic white, 18% non-Hispanic black, and 3% other (including multicultural). Using population-based sample weights, this was equivalent to a population of 179,557,944 adults 45.5 ± 0.6y with sex distribution 49% male and 51% female, and race distribution 7% Mexican-American, 6% other Hispanics, 73% non-Hispanic white, 10% non-Hispanic black, and 4% other. There were 114 subjects in this sample with a self-report of coronary heart disease (CHD) and 91 with a self-report of stroke, equivalent to less than 3% of this population with CHD or stroke.

Differences in physiological variables by fasting glucose status are presented in Table 1. Age, tHcy, glucose, HbA_1c_, insulin, BMI, and systolic blood pressure were significantly higher in IFG and DFG groups than the NFG group (*Ps *< 0.017). In addition, total cholesterol and serum creatinine were higher (*Ps *< 0.017) in IFG than NFG. Furthermore, age, glucose, HbA_1c_, insulin, BMI, and systolic blood pressure were higher in DFG than IFG (*Ps *< 0.017). There were no differences (*P *> 0.05) among groups with respect to vitamin status (serum folate and B_12_) and C_Cr_, although the comparison between DFG and IFG with respect to C_Cr _just missed significance (*P *= 0.02, adjusted α = 0.17).

**Table 1:** Select physiologic variables by fasting plasma glucose status among 3,485 U.S. adults (≥ 20y) with no self-reported diabetes.

**Variable**	**NFG (*n *= 2,244)**	**IFG (*n *= 1,108)**	**DFG (*n *= 133)**
**tHcy (μmol/L)**	7.4 [7.3–7.5]	8.3 [8.1–8.5]*	8.6 [8.0–9.2]†
**Age (years)**	42.3 ± 0.6	52.1 ± 0.7*	58.2 ± 2.3†‡
**Glucose (mg/dL)**	90.8 ± 0.2	106.9 ± 0.2*	170.8 ± 8.5†‡
**HbA_1c _(%)**	5.2 ± 0.0	5.5 ± 0.0*	7.2 ± 0.3†‡
**Insulin (uU/mL)**	9.8 ± 0.1	14.1 ± 0.4*	26.4 ± 1.9†‡
**BMI (kg/m^2^)**	26.7 ± 0.1	29.3 ± 0.2*	33.5 ± 1.3†‡
**SBP (mmHg)**	120.5 ± 0.6	127.5 ± 0.6*	135.7 ± 2.8†‡
**TC (mg/dL)**	198.9 ± 1.4	209.8 ± 1.6*	211.2 ± 5.9
**Folate (ng/mL)**	14.1 ± 0.3	14.5 ± 0.3	14.4 ± 0.9
**Vitamin B_12 _(pg/mL)**	492.2 ± 4.2	486.0 ± 8.5	486.5 ± 20.9
**Creatinine (μmol/L)**	68.4 ± 0.8	75.9 ± 2.4*	72.2 ± 2.9
**C_Cr _(mL/min)**	131.6 ± 1.9	126.5 ± 2.5	142.5 ± 10.4

Data are from the combined 1999–2000 and 2001–2002 National Health and Nutrition Examination Surveys. Non-standard abbreviations are NFG, normal fasting glucose, IFG, impaired fasting glucose, DFG, provisional diagnosis of diabetes, tHcy, total plasma homocysteine, SBP, systolic blood pressure, TC, total lipoprotein cholesterol, and C_Cr_, estimated creatinine clearance. Values are mean ± standard error, except for tHcy, which was log transformed because of its highly skewed distribution and is presented as median with 95% confidence interval (antilog values).

*P < 0.017 for NFG vs. IFG

† *P *< 0.017 for NFG vs. DFG

‡ *P *< 0.017 for IFG vs. DFG

Correlations between tHcy and physiologic variables, by fasting glucose status, are shown in Table 2. In the total sample, age (*r *= 0.41) and C_Cr _(*r *= -0.38) were the strongest correlates of tHcy (*Ps *< 0.0001). This was consistent when correlates of tHcy were examined by individual groups, with correlations for age ranging from *r *= 0.38 to 0.44, and for C_Cr _ranging from *r *= -0.35 to -0.46 (*Ps *< 0.001). Glucose and HbA_1c _were weaker, but still significant, correlates of tHcy in all subjects (*r *= 0.12 and 0.10, *Ps *< 0.0001), and in NFG (*r *= 0.11 and 0.12, *Ps *< 0.0001) and IFG groups (*r *= 0.15 and 0.07, *Ps *< 0.05). In contrast to the other groups, there was a negative relationship between tHcy and glucose (*r *= -0.14), and tHcy and HbA_1c _(*r *= -0.19), with only the latter being significant (*P *< 0.05).

**Table 2:** Relationships between total plasma homocysteine and select physiologic variables, by fasting glucose status, among 3,485 U.S. adults (≥ 20y) with no self-reported diabetes.

**Group**	**Age**	**Glucose**	**HbA_1c_**	**Insulin**	**BMI**	**SBP**	**TC**	**Folate**	**B_12_**	**S_Cr_**	**C_Cr_**
**Total**	0.41*	0.12*	0.10*	-0.00	-0.01	0.28*	0.11*	-0.10*	-0.25*	0.35*	-0.38*
**NFG**	0.38*	0.11*	0.12*	-0.05‡	-0.02	0.30*	0.14*	-0.15*	-0.24*	0.34*	-0.35*
**IFG**	0.38*	0.15*	0.07‡	-0.06	-0.07‡	0.19*	-0.01	-0.05	-0.26*	0.32*	-0.42*
**DFG**	0.44*	-0.14	-0.19‡	-0.21‡	-0.19‡	0.13	0.05	0.15	-0.28†	0.55*	-0.46*

Data are from the combined 1999–2000 and 2001–2002 National Health and Nutrition Examination Surveys. Non-standard abbreviations are NFG, normal fasting glucose, IFG, impaired fasting glucose, DFG, provisional diagnosis of diabetes, SBP, systolic blood pressure, TC, total lipoprotein cholesterol, S_Cr_, serum creatinine, and C_Cr_, estimated creatinine clearance. Values are Pearson correlation coefficients (*r*) using log transformed total homocysteine (log tHcy) as the dependent variable.

*P < 0.0001

†*P *< 0.01

‡*P *< 0.05

Results from univariate and multivariate regression models are presented in Table 3. Fasting glucose status was a significant predictor of tHcy (Model 1, *Ps *< 0.001). However, after adjusting for demographic and physiologic variables that influence homocysteine metabolism and/or fasting tHcy concentrations, fasting glucose status was no longer a significant predictor (Models 2 and 3, *Ps *> 0.05). Next, fasting glucose status was examined after adjusting for age, sex, and a measure of kidney function only. Similar to previous findings, fasting glucose status was not a significant predictor when serum creatinine was included in the model (Model 4, *Ps *> 0.05). However, fasting glucose status was a significant predictor when C_Cr _was included in the model (Model 5, *Ps *< 0.05). HbA_1c _was a significant predictor of tHcy in the model using C_Cr _as the measure of kidney function (Model 3, *P *< 0.05), but not in the model using serum creatinine (Model 2, *P *> 0.05).

**Table 3:** Univariate and multivariate linear models predicting total plasma homocysteine among 3,485 U.S. adults (≥ 20y) with no self-reported diabetes.

	**IFG**	**DFG**	**Sex**	**Age**	**HbA_1c_**	**Insulin**	**BMI**	**SBP**	**TC**	**Folate**	**B_12_**	**S_Cr_**	**C_Cr_**
**1**	0.1181*	0.1462†											
**2**	0.02	0.08	0.1143*	0.0077*	-0.03	-0.00	-0.00	0.0012‡	0.00	-0.0010*	-0.0003*	0.0020‡	
**3**	0.02	0.08	0.1598*	0.0047*	-0.0307§	-0.00	0.0070‡	0.0014†	0.00	-0.0010*	-0.0003*		-0.0021*
**4**	0.01	0.01	0.1391*	0.0068*								0.0019‡	
**5**	0.0326‡	0.0551§	0.1738*	0.0051*									-0.0012*

Data are from the combined 1999–2000 and 2001–2002 National Health and Nutrition Examination Surveys. Non-standard abbreviations are NFG, normal fasting glucose, IFG, impaired fasting glucose, DFG, provisional diagnosis of diabetes, SBP, systolic blood pressure, TC, total lipoprotein cholesterol, S_Cr_, serum creatinine, and C_Cr_, estimated creatinine clearance. Values are regression coefficients (β) from univariate and multivariate linear models using log transformed total homocysteine (log tHcy) as the dependent variable. For simplicity, the intercept term was omitted from the table but was significant (P ≤ 0.0001) in all models. Normal fasting glucose and female sex were used as reference groups.

*P < 0.0001

† *P *< 0.001

‡ *P *< 0.01

§*P *< 0.05

The analyses completed on subjects by self-report diabetes history included a sample of 275 adults, with an equal sex distribution, 34% Mexican-American, 7% other Hispanics, 36% non-Hispanic white, 19% non-Hispanic black, and 4% other (including multicultural). Using population-based sample weights, this was equivalent to a population of 12,322,266 adults 56.5 ± 1.1y with sex distribution 45% female and 55% male, and race distribution roughly 8% Mexican-American, 9% other Hispanics, 62% non-Hispanic white, 13% non-Hispanic black, and 8% other. Thirteen subjects (5% of this population) reported taking insulin, and 205 subjects (67%) reported use of diabetic pills to lower blood sugar. There were 28 subjects in this sample with a self-report of CHD and 13 with a self-report of stroke (about 8% of this population had CHD and 3.6% had stroke). The median tHcy level was 8.3 [7.9–8.6] μmol/L, and the average physiologic values were the following: fasting plasma glucose 154.6 ± 3.6 mg/dL, HbA_1c _7.2 ± 0.1%, fasting insulin 20.7 ± 1.7 uU/mL, BMI 31.4 ± 0.8 kg/m^2^, systolic blood pressure 131.7 ± 1.2 mmHg, total cholesterol 199.2 ± 2.6 mg/dL, serum folate 15.2 ± 0.8 ng/mL and vitamin B_12 _548.3 ± 22.4 pg/mL, serum creatinine 73.6 ± 3.2 μmol/L, and C_Cr _138.7 ± 7.3 mL/min.

Similar to findings among non-diabetic subjects, age (*r *= 0.41) and C_Cr _(*r *= -0.46) were the strongest correlates of tHcy (*Ps *< 0.0001), whereas glucose and HbA_1c _were weaker, but still significant, correlates of tHcy (*r *= -0.15 and -0.17, *Ps *< 0.05). Finally, results from univariate and multivariate regression models among subjects with a self-report history of diabetes are presented in Table 4. In the full models (1 and 2), measures of kidney function (either serum creatinine or C_Cr_) were again the strongest predictors of tHcy, whereas neither HbA_1c _nor insulin were significant predictors of tHcy in these models. In contrast, in the abbreviated models controlling for sex, age, and a measure of kidney function (models 3 and 4), serum creatinine was (*P *< 0.05), but C_Cr _was not, a significant predictor of tHcy.

**Table 4:** Univariate and multivariate linear models predicting total plasma homocysteine among 275 U.S. adults (≥ 20y) with a self-report history of diabetes.

	**Sex**	**Age**	**HbA**_1c_	**Insulin**	**BMI**	**SBP**	**TC**	**Folate**	**B_12_**	**S_Cr_**	**C_Cr_**
**1**	-0.07	0.0114*	-0.01	-0.00	0.00	0.00	-0.00	-0.00	-0.0004‡	0.0025†	
**2**	-0.1139†	0.0058‡	-0.01	-0.00	0.0154†	0.00	-0.00	-0.00	-0.00		-0.0022†
**3**	-0.07	0.0094*								0.0021‡	
**4**	-0.0979‡	0.0074†									-0.00

Data are from the combined 1999–2000 and 2001–2002 National Health and Nutrition Examination Surveys. Non-standard abbreviations are SBP, systolic blood pressure, TC, total lipoprotein cholesterol, S_Cr_, serum creatinine, and C_Cr_, estimated creatinine clearance. Values are regression coefficients (β) from univariate and multivariate linear models using log transformed total homocysteine (log tHcy) as the dependent variable. For simplicity, the intercept term was omitted from the table but was significant (P ≤ 0.0001) in all models. Female sex was used as the reference group.

*P < 0.0001

† *P *< 0.01

‡ *P *< 0.05

## Discussion

This study demonstrates that fasting tHcy levels are elevated in non-diabetic subjects as a function of fasting glucose status; tHcy concentrations increased progressively with worsening fasting glucose. Subjects with self-reported diabetes had levels similar to levels found in non-diabetic subjects who had abnormally high fasting gluocse levels. The difference in fasting tHcy was 1.2 μmol/L between groups representing the extremes of fasting glucose, and 0.9 μmol/L between subjects with a self-report history of diabetes and those without a self-report history of diabetes and normal fasting glucose. The clinical significance of such small differences is unknown, particularly in light of the fact that tHcy levels were within the normal range (less than 15 μmol/L) in all groups of subjects.

Although fasting tHcy levels differed as a function of fasting glucose status, results from correlation and multivariate regression analyses were in agreement that age and kidney function (measured as serum creatinine or estimated C_Cr_) were the major correlates of fasting tHcy concentrations among non-diabetic and diabetic individuals. In contrast, these analyses provided much less support for the concept that glucose metabolism has a major influence on fasting tHcy concentrations.

These findings were somewhat surprising, based on previous reports demonstrating that fasting tHcy levels were related to metabolic control in patients with type 1 and type 2 diabetes [13-16]. In a clinic-based study [13], fasting tHcy levels were nearly doubled in poorly-controlled than well-controlled patients with type 2 diabetes. In a prospective study [14], fasting tHcy levels decreased in patients with type 2 diabetes who experienced modest improvements in metabolic control, whereas fasting tHcy increased in patients who had worsened metabolic control over follow-up. Because measures of kidney function (blood urea nitrogen and serum creatinine) and vitamin status (serum folate and B_12_) were within normal clinical limits at baseline and remained unchanged during follow-up, these findings imply a direct effect of metabolic control on fasting tHcy. However, in the present study HbA_1c _was only weakly correlated with fasting tHcy in non-diabetic subjects, irrespective of fasting glucose status. The relationship between HbA_1c _and tHcy was highest in non-diabetic subjects with normal fasting glucose, and was negative in those with a provisional diagnosis of diabetes, which was opposite to the expected direction. Similarly, HbA_1c _was weakly and negatively correlated with tHcy in diabetic subjects. In multivariate regression analyses, age and measures of kidney function (serum creatinine or C_Cr_) were the major predictors of fasting tHcy in non-diabetic and diabetic subjects, whereas HbA_1c _was not a significant predictor in either group of subjects.

One factor that might explain why the present results differ from others with respect to the role of metabolic control on fasting tHcy is that our study includes current data obtained from a broad cross-section of the U.S. population, whereas other studies were conducted in The Netherlands [9-11], Poland [13], and Italy [14]. Differences in race and/or lifestyle of individuals living in the various countries represented in these studies may have contributed to the differential results. In addition, the U.S. instituted a program of cereal grain fortification in 1998, whereas the other countries listed do not have a fortification practice in place, which could have attenuated fasting tHcy levels in subjects from the U.S.

The present findings were also somewhat surprising in that fasting insulin had little influence on fasting tHcy levels. Although relationships between fasting insulin and fasting tHcy were negative in non-diabetic subjects, which is in the expected direction, the strength of these correlations was weak, and were essentially non-existent in diabetic subjects (data not shown). Furthermore, fasting insulin was not a significant predictor in any model of fasting tHcy. This is somewhat in contrast to findings in which acute hyperinsulinemia led to a decrease in tHcy in normal subjects, but not in insulin resistant type 2 diabetes patients, suggesting that insulin had a lowering effect on tHcy, except in the case of patients who were resistant to this effect of insulin [20]. On the other hand, in support of the present findings a recent report [21] failed to show any influence of insulin resistance or degree of metabolic control on fasting tHcy levels in obese patients with type 2 diabetes patients treated with daily insulin over a one-month period.

Although the findings with respect to metabolic control and insulin were somewhat surprising and in contrast to other studies, what was not surprising was that kidney function played a major role in modulating fasting tHcy concentrations. Because many of the studies mentioned previously only included a single measure of serum creatinine, used as a surrogate marker of renal function, estimated C_Cr _was included in the present analyses to provide a better assessment of the impact of kidney function on fasting tHcy levels. The rationale for this was that an estimate of glomerular filtration rate is the best measure of overall kidney function in health and disease [22]. Furthermore, the kidney has substantial tHcy handling and metabolizing capabilities [23] and thus it is possible that previous studies did not adequately account for differences in kidney function by using serum creatinine alone.

The results of the present study support this contention. For example, estimated C_Cr _was a slightly stronger correlate of fasting tHcy than serum creatinine alone in both diabetic and non-diabetic subjects. In addition, estimated C_Cr _was a slightly stronger predictor of fasting tHcy in multivariate models. This is in contrast to a recent report [24] that fasting tHcy concentrations were closely and independently associated with estimated glomerular filtration rate, but not with serum creatinine, in groups of patients with type 1 and type 2 diabetes undergoing intensive insulin treatment. On the other hand, the present results are supported by previous studies, including Stabler and colleagues [25], and Davies and colleagues [26], who demonstrated the importance of renal function on modulating tHcy levels, at least in patients with type 2 diabetes. The present results also suggest that there is a differential impact of C_Cr_, compared to serum creatinine, on fasting tHcy levels. In non-diabetic subjects, fasting glucose status was a significant predictor of tHcy after adjusting for age, sex, and C_Cr_, but not when serum creatinine was substituted for C_Cr_. Likewise, sex and age, but not C_Cr_, were significant predictors of tHcy in the abbreviated models (see Models 3 and 4 in Table 4), whereas age and serum creatinine, but not sex, where significant predictors of tHcy when serum creatinine was substituted for C_Cr _in the models.

Some factors inherent to the study design may have contributed to the differential findings in this study compared to others, including differences in groups with respect to demographic and health status variables. For example, among non-diabetic subjects, the normal fasting glucose group had more females than males (~ 56% vs. 44%), whereas both the impaired fasting glucose and provisional diagnosis of diabetes groups had more males than females (~ 60% vs. 40% in both groups). This could have contributed to the higher tHcy levels in the latter two groups because fasting tHcy is typically higher in men than women [27,28]. Furthermore, sex was a significant predictor of fasting tHcy in all regression models in non-diabetic subjects.

In light of the differences in sex distribution, separate regression models were run for women and men to determine if there were differences in major correlates of fasting tHcy according to sex. Among females, in the full regression model using serum creatinine, age, insulin, total cholesterol, folate, B_12_, and creatinine were significant predictors of fasting tHcy. Among males, age, HbA_1c_, systolic blood pressure, folate, and B_12_, but not creatinine, were significant predictors of fasting tHcy. Among females, in the full regression model using C_Cr_, age, BMI, folate, B_12_, and C_Cr _were significant predictors of tHcy. Among males, age, HbA_1c_, systolic blood pressure, folate, B12, and C_Cr _were significant predictors of tHcy. Differences between the sexes with respect to major correlates of tHcy were also apparent in the abbreviated regression models. Among females, age and creatinine were both significant predictors of tHcy. Among males, age, but not creatinine, was a significant predictor of tHcy. Among females, the overall effect of fasting glucose status, age, and estimated C_Cr _were significant predictors of tHcy. Among males, fasting glucose status was not a significant predictor of tHcy, after adjusting for age and estimated C_Cr _(both terms were significant).

Second, there were some differences with respect to self-reported health status among non-diabetic subjects. Roughly 2% of non-diabetic subjects with normal fasting glucose had a self-report of CHD, whereas 4% of non-diabetic subjects with impaired fasting glucose and 9% with a provisional diagnosis of diabetes reported CHD. The same pattern emerged for stroke (about 2%, 3%, and 7% for normal, impaired, and provisional diagnosis of diabetes groups). Because macroangiopathy [12] has been shown to impact fasting tHcy, at least in patients with type 2 diabetes, differences in the prevalence of CHD and stroke may have contributed to the group differences in tHcy. However, these differences were not so large that self-reported health status alone would likely account for differences in fasting tHcy levels among the various groups of non-diabetic subjects.

The cross-sectional nature of NHANES does not allow for the determination of the underlying cause(s) of the elevated fasting tHcy levels in non-diabetic subjects with impaired fasting glucose/a provisional diagnosis of diabetes, or in diabetic subjects. However, results from the various analyses were consistent in demonstrating the importance of age and kidney function in modulating fasting tHcy levels, while providing much less support for an important role of glucose metabolism in this regard. The results of this study were also fairly consistent with those of Russo and colleagues [29], who found that age, creatinine, vitamin status, sex, smoking, methylene tetrahydrofolate reductase (MTHFR) genotype, and systolic blood pressure were significantly associated with tHcy in a clinic-based study of Italian adults with type 2 diabetes. Similarly, that study found no significant associations between tHcy and diabetes-related variables, such as diabetes duration, fasting glucose, HbA_1c_, current treatment regimen, and diabetes complications. Although data on MTHFR genotype was not available in NHANES, it is unlikely that large differences existed with respect to genotype distribution among the various groups because the data were drawn from a large, stratified random sample. However, this possibility cannot be entirely ruled out.

## Conclusion

This study supports the concept that age and kidney function, and not measures of glucose metabolism (fasting glucose, level of metabolic control, and insulin), are the primary correlates of fasting tHcy levels among non-diabetic and diabetic adults in the U.S.

## Competing interests

The author(s) declare that they have no competing interests.

## Authors' contributions

GED conceived the study, participated in its design and analysis, and drafted the manuscript. SML and XHZ performed the statistical analyses and edited the manuscript. All authors read and approved the final manuscript.

## Note

http://www.cdc.gov/nchs/about/major/nhanes/datalink.htm#1999%20Current%20NHANES
